# 836 Imaging Technologies for Clinical Assessment of Burns and Prediction of Healing Outcomes: A Systematic Review

**DOI:** 10.1093/jbcr/iraf019.367

**Published:** 2025-04-01

**Authors:** Zachery Harris, Karin Hasegawa, Emily Peterson, Arash Karimi, Erica Heller, Abdullah Ashrafi, Kuangyi Xu, Steven Sandoval, M Hassan Arbab

**Affiliations:** Stony Brook University; Stony Brook University; Stony Brook University; Stony Brook University; Stony Brook University; Stony Brook University; Stony Brook University; Stony Brook University; Stony Brook University

## Abstract

**Introduction:**

An accurate and precise method of burn depth classification is essential for making appropriate burn treatment decisions. Surgical excision and grafting are classically performed for deeper burns (e.g. deep partial-thickness, full-thickness) which also involve increased scarring and infection risk. Clinical distinction of superficial versus deep partial-thickness burns, however, is not entirely accurate.

**Methods:**

This presentation discusses the results of a systematic literature review on the imaging technologies studied for in vivo burn wound depth classification. It was based on the preferred reporting items for systematic reviews and meta-analyses (PRISMA) guidelines. Reported measures of accuracy, resolution, penetration depth, and scanning speed were recorded.

**Results:**

The review included 259 studies—published between 1987 and 2024—about 18 unique modalities. These modalities distinguished burn wound depth based on tissue perfusion, edema formation, structural changes in collagen and vascular networks, oxygen saturation, and more. Evidence suggests that some technologies may be used to accurately assess burn wound depth to varying degrees. For instance, laser doppler imaging is based on the foundational finding of reduced flux in deep partial- and full-thickness burns. Other technologies of interest explored more recently include spectral imaging, such as terahertz, near infrared, and multi-/hyperspectral imaging, spatial frequency domain imaging, and optical coherence tomography. Recent efforts in machine learning and artificial intelligence-based methods are also surveyed. Limitations may include imaging speed, field of view, lack of standardization, and insufficient accuracy.

**Conclusions:**

This presentation will educate medical professionals on the biological contrast mechanism relevant to skin burns captured by each of these imaging modalities, as well as accuracy measures, relevant clinical applications, and current limitations. A meta-analysis using data collected from the reviewed publications will be presented.

**Applicability of Research to Practice:**

An accurate and precise method of burn depth classification is essential for making appropriate burn treatment decisions.

**Funding for the Study:**

This work was supported in part by the U.S. Army Medical Research Acquisition Activity (USAMRAA) through the Military Burn Research Program (MBRP) under Award No. W81XWH-21-1-0258, and the National Institute of General Medical Sciences (GM112693).

